# Memory deficits in amyotrophic lateral sclerosis are not exclusively caused by executive dysfunction: a comparative neuropsychological study of amnestic mild cognitive impairment

**DOI:** 10.1186/1471-2202-15-83

**Published:** 2014-06-30

**Authors:** Judith Machts, Verena Bittner, Elisabeth Kasper, Christina Schuster, Johannes Prudlo, Susanne Abdulla, Katja Kollewe, Susanne Petri, Reinhard Dengler, Hans-Jochen Heinze, Stefan Vielhaber, Mircea A Schoenfeld, Daniel M Bittner

**Affiliations:** 1 German Center for Neurodegenerative Diseases (DZNE), Magdeburg, Leipziger Straße 44, 39120 Magdeburg, Germany; 2 Department of Neurology, Otto-von-Guericke University, Leipziger Straße 44, 39120 Magdeburg, Germany; 3 German Center for Neurodegenerative Diseases (DZNE), Rostock, Gehlsheimer Straße 20, 18147 Rostock, Germany; 4 Department of Neurology, Hannover Medical School, Carl-Neuberg-Str. 1, 30625 Hannover, Germany; 5 Leibniz Institute for Neurobiology, Brenneckestraße 6, 39118 Magdeburg, Germany; 6 Kliniken Schmieder, Zum Tafelholz 8, 78476 Allensbach, Germany

**Keywords:** Cognitive neuropsychology, ALS, Episodic memory

## Abstract

**Background:**

Recent work suggests that ALS and frontotemporal dementia can occur together and share at least in part the same underlying pathophysiology. However, it is unclear at present whether memory deficits in ALS stem from a temporal lobe dysfunction, or are rather driven by frontal executive dysfunction. In this study we sought to investigate the nature of memory deficits by analyzing the neuropsychological performance of 40 ALS patients in comparison to 39 amnestic mild cognitive impairment (aMCI) patients and 40 healthy controls (HC). The neuropsychological battery tested for impairment in executive functions, as well as memory and visuo-spatial skills, the results of which were compared across study groups. In addition, we calculated composite scores for memory (learning, recall, recognition) and executive functions (verbal fluency, cognitive flexibility, working memory). We hypothesized that the nature of memory impairment in ALS will be different from those exhibited by aMCI patients.

**Results:**

Patient groups exhibited significant differences in their type of memory deficit, with the ALS group showing impairment only in recognition, whereas aMCI patients showed short and delayed recall performance deficits as well as reduced short-term capacity. Regression analysis revealed a significant impact of executive function on memory performance exclusively for the ALS group, accounting for one fifth of their memory performance. Interestingly, merging all sub scores into a single memory and an executive function score obscured these differences.

**Conclusion:**

The presented results indicate that the interpretation of neuropsychological scores needs to take the distinct cognitive profiles in ALS and aMCI into consideration. Importantly, the observed memory deficits in ALS were distinctly different from those observed in aMCI and can be explained only to some extent in the context of comorbid (coexisting) executive dysfunction. These findings highlight the qualitative differences in temporal lobe dysfunction between ALS and aMCI patients, and support temporal lobe dysfunction as a mechanism underlying the distinct cognitive impairments observed in ALS.

## Background

Amyotrophic lateral sclerosis (ALS) is a progressive neurodegenerative disorder, characterized by hallmark motor neuron and corticospinal tract degeneration. It is well-established that ALS shares clinical [1], pathological [2] and genetic [3] characteristics with frontotemporal dementia (FTD), and this overlap has led some studies to assert the existence of a single continuum on which both ALS and FTD lie [4,5]. Recent studies indicate a global neuropsychological deficit wherein impairment of executive functions is reported the most [6-14]. However, there is increasing evidence that other cognitive domains such as language and memory are also affected [7,10,15-19]. Especially memory impairment has been somewhat neglected and often seen as a failure of encoding as an executive component of memory [8,17,20]. However, there are reports on memory dysfunction in ALS, often tested with picture recall, word list learning, pair association learning or story recall [12,14,18,21-30]. Most of them support an encoding or short recall deficit with relatively sparing of consolidation performance but the findings are inconsistent and have not been related to temporal lobe dysfunction. Recently, there has been growing postmortem and in vivo evidence of temporal lobe involvement in ALS, based on hippocampal TDP-43 pathology [31,32] and volume loss [33-35]. Temporal lobe pathology is also a key feature of Alzheimer's Disease (AD) with hippocampal atrophy even in early stages [36]. Based on the shared hippocampal involvement in both ALS and AD, we sought to investigate whether there are distinctive differences between the sort of memory deficits in ALS and AD. However, the extent of hippocampal pathology in AD is, up to current knowledge, far more progressed and widespread than in ALS making a direct comparison between ALS and AD patients very difficult if not impossible. Therefore we decided to compare ALS patients to a group of patients that suffer from an AD prodromal stage, amnestic mild cognitive impairment (aMCI) [37]. Patients with aMCI have overt deficits in cognition, especially in the memory domain, but are still largely independent in daily activities. They have a far higher risk of developing dementia than cognitively normal persons, but at the individual patient level the prognosis might be variable [38]. The temporal lobe pathology as hippocampal atrophy is also a hallmark in aMCI but it occurs to a much smaller extent than in AD [39]. Therefore this group of patients is comparable to patients with ALS in terms of hippocampal degeneration. In the current work we focussed on verbal memory performance and hypothesized a qualitative difference between patients with ALS and aMCI.

## Methods

### Subjects

40 ALS patients were recruited from the outpatient clinics of the Departments of Neurology at the Otto-von-Guericke University of Magdeburg and Hannover Medical School between 04/2011 and 03/2012. ALS patients were diagnosed according to the revised El Escorial Criteria [40], and the Amyotrophic Lateral Sclerosis Functional Rating Scale - Revised (ALSFRS-R, [41]) was used to evaluate their functional status. Three ALS patients fulfilled the Rascovsky criteria for behavioural variant frontotemporal dementia (ALS-FTD, [42]), and were included in the analysis to represent the full spectrum of cognitive impairment in ALS. ALS Patients with a history of other neurological conditions that could affect cognition (major hemispheric stroke, traumatic brain injury), severe active mental illness, or alcohol abuse were excluded.

In addition, 39 aMCI patients were recruited from the memory outpatient clinic in Magdeburg and classified according to the revised Peterson Criteria for mild cognitive impairment [37]. A group of 40 healthy age- and gender- matched controls without a prior history of neurological or psychiatric illness was also recruited. In the healthy control group, only subjects who performed within the normal range of the Montreal Cognitive Assessment (MoCA; cut-off 26/30) were included. The local ethics committee of Otto-von-Guericke University approved the study and all participants gave their written informed consent prior to their inclusion.

### Neuropsychological assessment and data analysis

All participants underwent a detailed neuropsychological assessment in conjunction with their clinical examination. The battery of tests included a range of standardized neuropsychological instruments and was designed based on the ALS literature suggesting an early decline of frontally-mediated executive functions in ALS patients [6-8,10]. To assess the executive domain, the Regensburger verbal fluency test [43] was used to survey phonemic verbal fluency (letter “K”) and flexibility (alteration between letters “G” and “R”). Additionally, we tested for cognitive flexibility with the Trail Making Test (the ratio between part B and A was computed to account for motor impairment) [44] and for verbal working memory performance (Wechsler Memory scale-revised (WMS-R), digit span backwards) [45]. The battery also tested for impairment in non-executive cognitive domains. To assess memory functions, the German version of the Rey Auditory Verbal Learning Test (RAVLT) [46], and the forward digit span task from the revised Wechsler Memory Scale [45] was used. Visuo-spatial abilities were evaluated using the Rey Complex Figure Test (RCFT) [47]. Mood was assessed using Beck Depression Inventory-II [48]. Neuropsychological performance between the study groups was compared by one-way analysis of variance (ANOVA) with group as a main factor (ALS/aMCI/HC). Because of some physical disabilities in the ALS cohort, not all patients completed all tests. The missing data strategy was to exclude them from individual analyses but the cases were retained in the dataset. All tests were two-tailed and the statistical significance threshold was set at p < 0.05. Bonferroni correction was applied to adjust the α-value for post hoc analysis where applicable.

To determine whether the patient groups could be differentiated based on group level with less refined neuropsychological measures, we computed composite scores for “Memory” and “Executive function” by transforming the raw values into Z-scores that were referenced by mean and standard deviation of the healthy controls on the corresponding test. Composite “Memory” scores incorporated the following parameters: learning (sum of trials 1–5 of the RAVLT), immediate verbal recall (difference between trial 5 and trial 6), delayed verbal recall (difference between trial 5 and trial 7) and recognition (corrected for false positive and interference items). “Executive function” score included measures of verbal fluency, cognitive flexibility and verbal working memory. These scores were compared across patient groups (ALS/aMCI) using t-tests for non-dependent samples, but also used to conduct a regression analysis to estimate the influence of executive function on memory performance in each of the three groups.

All statistical analyses were carried out using SPSS for Windows, Version 21.0 (IBM SPSS Statistics, Armonk, NY, USA; RRID:rid_000042).

## Results

### Demographic and clinical characteristics

Demographic and clinical data are summarized in Table 1. An analysis of variance indicated no difference in age (F_2,116_ = 1.92, p = 0.15) and a trend in gender distribution (*Χ*^2^ = 5.53, p = 0.06). There was also a trend toward higher self-reported depression scores in ALS patients (F_2,103_ = 2.94, p = 0.06). However, none of the three groups scored within the range of clinically relevant depressive symptoms (Beck Depression Inventory-II <13 points). There was a difference between groups regarding their completed educational years (F_2,114_ = 4.28, p = 0.02). Patients with ALS (13.0 ± 2.5) have less educational years than aMCI (14.6 ± 2.8), but both patient groups did not differ from healthy controls (14.0 ± 2.1).

**Table 1:** Baseline characteristics

**Demographic variable**	**ALS**	**N**	**Amnestic MCI**	**N**	**Healthy controls**	**N**	**p-value**
Age at time of assessment	60.4 (12.2)	40	64.5 (5.4)	39	62.7 (10.2)	40	0.152
Male sex	65.0% (-)	40	45.0% (-)	39	69.2% (-)	40	0.063
Education (years)*	13.0 (2.5)	40	14.6 (2.8)	37	14.0 (2.1)	40	**0.016**
BDI-II (estimated depression scores)	7.9 (6.6)	34	6.8 (4.7)	35	4.7 (4.9)	40	0.057
ALSFRS-R	38.4 (7.8)	40	n.a.		n.a.		n.a.
Disease duration (in months; from symptom onset)	22.16 (19.5)	40	n.a.		n.a.		n.a.
Disease progression	0.54 (0.36)	40	n.a.		n.a.		n.a.

Values are mean (SD). Two-tailed p-values of <0.05 are considered significant, shown in bold.

ALS, amyotrophic lateral sclerosis; MCI, mild cognitive impairment; BDI-II, Beck Depression Inventory-II; ALSFRS-R, ALS Functional Rating Scale Revised; n.a., not applicable; disease duration, time from symptom onset to assessment; disease progression, estimated using decline in ALS Functional Rating Scale (ALSFRS-R) score since symptom onset (48-ALSFRS-R/disease duration [38]); *Years of education include: Years attended in school plus years of the longest, completed education.

### Neuropsychological performance of ALS, aMCI patients, and control subjects

**Figure 1:**
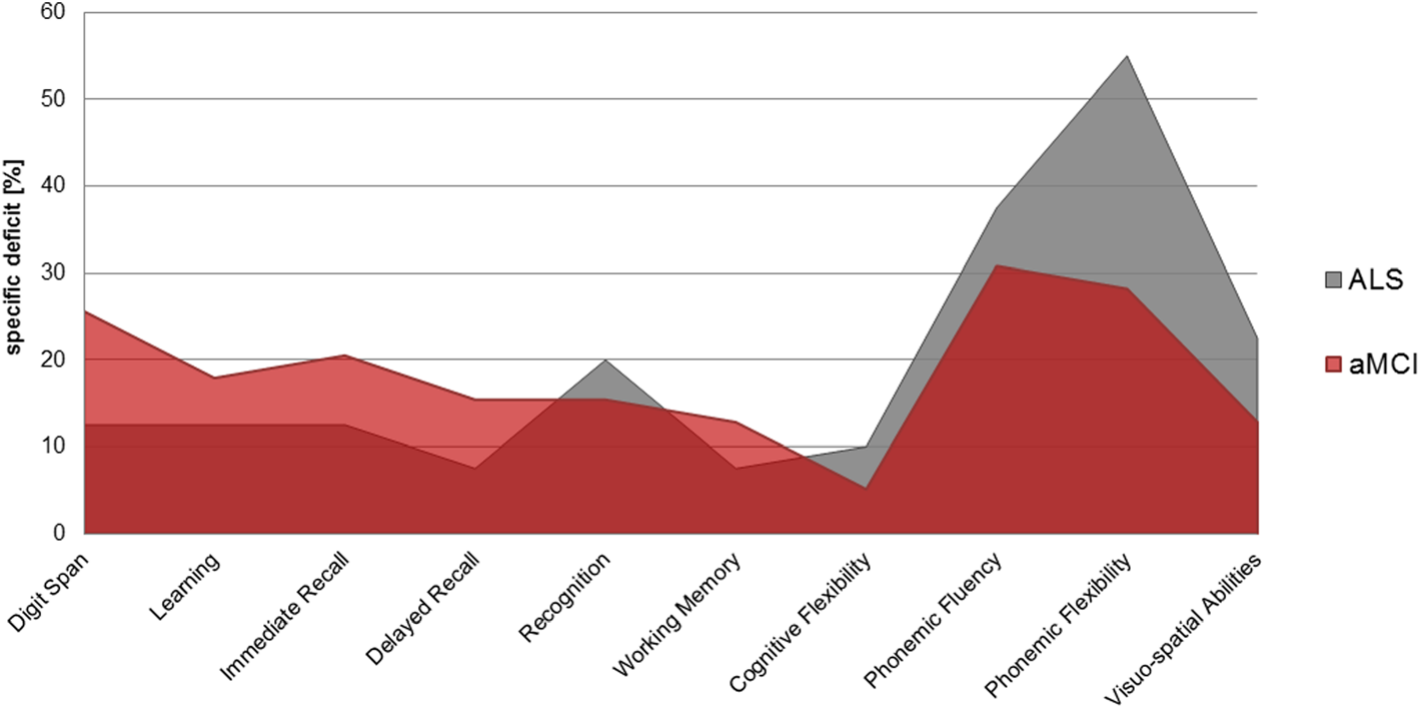
**Neuropsychological profile of patients with ALS and aMCI.** Profile lines display the percentage of ALS patients and aMCI patients <1.5 SD below the mean of healthy controls for subtests within each cognitive domain.

A significant group effect was identified in the memory domain for the following variables: Digit span (F_2,114_ = 8.58, p < 0.001), immediate verbal recall (F_2,115_ = 5.91, p = 0.004), delayed verbal recall (F_2,115_ = 6.97, p = 0.001) and recognition (F_2,116_ = 3.69, p = 0.028). Significant group effects were also revealed for tests of executive function: Phonemic fluency (F_2,112_ = 11.00, p < 0.001) and flexibility (F_2,112_ = 18.47, p < 0.001) and working memory performance (F_2,114_ = 10.48, p < 0.001). Visuo-spatial performance was also significantly different between the three groups (F_2,104_ = 6.42, p = 0.002). No differences were observed for verbal learning (F_2,116_ = 2.67, p = 0.074). Mean values and standard deviations for neuropsychological subtests are presented in Table 2. Figure 1 illustrates the percentage of aMCI patients and ALS patients that fell more than 1.5 standard deviations below the performance of healthy controls in each neuropsychological subtests.

**Table 2:** Post-hoc test results in neuropsychological tests for patients and controls: raw scores and p - values

	**ALS**	**N**	**aMCI**	**N**	**HC**	**N**	**p - value ALS vs. HC**	**p - value aMCI vs. HC**	**p - value ALS vs. aMCI**
**Executive functions**									
Phonemic fluency*	10.9 (4.5)	40	12.4 (4.1)	35	15.2 (4.0)	40	**<0.001**	**0.011**	0.39
Phonemic flexibility*	9.0 (3.3)	40	10.7 (3.5)	35	13.3 (2.9)	40	**<0.001**	**0.002**	0.08
Digit span backward raw score*	5.5 (1.5)	38	4.6 (1.2)	39	6.1 (1.4)	40	0.26	**<0.001**	**0.02**
Cognitive flexibility (TMT ratio)	2.8 (1.0)	34	2.6 (1.0)	38	2.5 (1.4)	40	0.62	1.00	1.00
**Memory**									
RAVLT Learning (∑1-5)	44.3 (10.5)	40	42.6 (8.2)	39	47.3 (9.0)	40	0.43	0.07	1.00
RAVLT Immediate recall (5–6)*	2.6 (1.7)	39	3.2 (1.8)	39	1.9 (1.7)	40	0.19	**0.003**	0.37
RAVLT Delayed recall 20’-30’ (5–7)*	2.7 (1.8)	40	3.7 (2.2)	39	2.0 (2.2)	39	0.41	**0.001**	0.08
RAVLT Recognition (W–F)*	9.1 (5.9)	40	9.4 (4.9)	39	11.8 (3.4)	40	**0.04**	0.09	1.00
Digit span forward raw score*	7.2 (1.5)	38	6.3 (1.3)	39	7.6 (1.5)	40	0.90	**<0.001**	**0.01**
**Visuospatial functions**									
RCFT*	31.0 (3.8)	34	33.4 (2.6)	33	33.1 (2.6)	40	**0.01**	1.00	**0.005**

Values are mean (SD). Two-tailed p values of <0.05 are considered significant, shown in bold.

ALS, Amyotrophic Lateral Sclerosis; aMCI, amnestic mild cognitive impairment; HC, healthy controls; RCFT, Rey Complex Figure Test; *significant on one-way ANOVA.

The analysis of composite scores for each cognitive domain revealed no main effect for overall “Memory” and “Executive function”. Within-group comparisons in the form of regression analysis revealed no significant impact of “Executive function” on “Memory” for neither the aMCI group (r = 0.25, p = 0.13) nor the healthy control group (r = -0.02, p = 0.88). In ALS patients there was a significant relationship between the two domains (r = 0.45, p = 0.003) with “Executive function” accounting for 20.5% of the variance in “Memory” performance.

## Discussion

This cross-sectional study of neuropsychological performance in controls, ALS and aMCI patients revealed a significant deficit in verbal memory function in both patient groups relative to controls. In patients with ALS we observed additional dominant executive deficits including the impairment of verbal fluency, which is a consistent, well-documented finding in motor neuron disease [7,8,11,14]. This deficit also has an impact on disease progression [6] and survival [49]. In contrast, reports on memory dysfunction are more inconsistent. When evaluating overall memory impairment as a composite parameter of several memory subdomains, there were no differences between aMCI and ALS patients. However, examining the nature of amnestic deficits based on more predefined sub-functions revealed disease-group specific patterns of impairment. Patients with aMCI had overt deficits in short and delayed recall, whereas ALS patients were mainly impaired in verbal memory recognition. Recall deficits in aMCI are a hallmark feature of this patient group and support the notion of being a pre-Alzheimer's Disease stage [37]. Recall performance in patients with ALS was not affected. Previous studies, that took verbal memory into account, have reported both, impaired and preserved recall performance on word-list learning tests, depicting the inconsistency among observed memory deficits in ALS. One study reported long-delay recall impairment but no impairment on short-term recall or recognition [23], while others report deficits in short recall performance but preserved delayed recall performance [12], or impairment in both, short and delayed recall [18]. Based on the high variability regarding cognitive deficits between patients, recent population-based studies propose a neuropsychological classification into subgroups with focus on executive impairment or non-executive impairment [7] and a novel screening tool has taken into account ALS specific and non-specific tests [15]. However, given the results yielded in the present study, memory deficits in ALS seem to be disease specific and can be differentiated from other neurodegenerative diseases such as AD when assessed specifically. Recognition deficits in patients with ALS were found the most obvious memory deficit and have been reported before [17,50], although such deficits have been mostly associated with executive dysfunction [17,29]. In our cohort, executive impairment could only account for 20.5% of memory performance in ALS, which supports the emerging notion that executive function is only one of several cognitive domains impaired in ALS [7,16]. Moreover, executive dysfunction in frontotemporal dementia has been related to failure in source memory but not recognition performance [51], which leads to the assumption that the recognition deficit reported here cannot be caused solely by executive dysfunction. Impairment in recognition can emerge from either insufficient encoding as a temporal lobe function or from deficient prefrontal cortical function. However, both assumptions would lead to a higher amount of errors and interference biases, which was more pronounced in patients with ALS than with aMCI. In experimental psychology, recognition memory is understand as a 'dual-process’ model that incorporates the product of two different memory functions, namely familiarity and recollection [52]. Thus, recognition judgements can be based on the recollection of details about previous events or on the assessment of stimulus familiarity [53]. In ALS, only one study so far investigated recognition memory and its underlying neuronal mechanisms [50]. The paradigm consisted of a verbal recognition task, where the subjects had to decide whether a word was presented before or not. Although this was an experimental set up, results can be compared to the recognition test that was used in this study (RAVLT). Similar to our observations, Muente et al. [50] reported a recognition deficit in patients with ALS that was related to an absent recognition associated event-related potential. Interestingly, this effect was not observed in patients with Alzheimer's Disease which completed a comparable task [54]. These results support the different qualities of memory impairment in ALS and AD observed in the current study.

In addition to detailed neuropsychological testing and/or neurophysiological measures, imaging studies could provide more information about the specific anatomical structures involved. A number of studies correlated cognitive performance with white or grey matter integrity [4,30,55,56], but only two focused on word list learning and structural integrity. The results however seem inconsistent since one reported a relationship between memory performance in ALS and uncinate fasciculus (UF) integrity [30], whereas the other study found no such association [55]. Since both studies had relatively small sample sizes, further structural-functional correlations are needed to draw more definite conclusions on the relationship between memory impairment and cerebral pathology in ALS. Given that the uncinate fasciculus connects temporal lobe structures such as the hippocampus with frontal lobe areas, its involvement highlights the contribution of structures other than the frontal lobe in memory performance in ALS. Hippocampal and parahippocampal pathology in ALS are well-described in post mortem studies [32,34,35], and lesions have been related to memory deficits. Interestingly, those lesions were different from those found in Alzheimer's Disease [34], which underlines the distinct neuropsychological profiles between patients with ALS and aMCI in the present study. There is further evidence from structural imaging, that the hippocampus, a key structure in memory function, is affected in ALS. Both hippocampal volume reduction [33] and parahippocampal surface alterations [57,58] are sites of ALS pathology, but these changes have not been related in-vivo to patients’ memory performance to date. Overall, imaging data support the notion that memory impairment in ALS is not caused exclusively by executive dysfunction. Further research needs to establish the relationship of temporal lobe involvement and memory impairment in ALS.

This study measured a wide range of cognitive domains, including verbal memory, executive functions and visuo-spatial skills with standardized neuropsychological tests. Tests of verbal memory revealed substantial recognition deficits in patients with ALS. However, there are some limitations to take into account. Initially we did not have a specific hypothesis which sub-function of memory would differ between ALS and aMCI and therefore chose a memory test that covers several sub-functions (RAVLT). With the results presented here, further research should focus on recognition memory, both verbal and visual, and investigate if the observed deficits are caused by deficient familiarity or recollection performance. It would be of interest to relate it to prefrontal lobe dysfunction as well, either measured by behavioural or imaging parameters in order to identify correlates of impaired recognition in ALS.

## Conclusions

In conclusion, the current investigation suggests that memory impairment in ALS is different from that observed in Alzheimer's disease, but also that there is considerable overlap in verbal memory performance between these conditions. The differentiation between the distinct cognitive profiles of the two patient groups can only be captured with detailed subdomain specific neuropsychological testing, and composite scores of domain level performance proved insufficient. Moreover, this study further underscores on the considerable extramotor deficits in ALS that extends well beyond executive dysfunction. Emerging cognitive screening tests in ALS, which are increasingly used in clinical trials and specialist clinics should take the unique memory deficits of ALS into consideration. The pragmatic implications of memory impairment in ALS in relation to compliance with medications and assistive devices are currently also understudied. At present there is a growing consensus that cognitive impairment in ALS is not solely dominated by executive dysfunction, but language and amnestic deficits are equally important.

## Abbreviations

ALS: Amyotrophic lateral sclerosis; aMCI: Amnestic mild cognitive impairment; FTD: Frontotemporal dementia; TDP-43: TAR DNA-binding protein 43; AD: Alzheimer's disease; ALSFRS-R: ALS functional rating scale-revised.

## Competing interests

The authors declare that they have no competing interest.

## Authors’ contributions

JM carried out data collection and analysis and drafted the manuscript. VB carried out data collection and analysis and revised the manuscript for intellectual content. EK, CS and SA participated in data collection and analysis. JP, KK, SP, RD and HH conceived of the study and participated in its design and coordination. SV supervised all clinical aspects of the study and participated in its design and coordination and revised the manuscript for intellectual content. MS participated in study design and coordination and helped drafting the manuscript. DB conceived of the study, and participated in its design and coordination and helped to draft the manuscript. All authors read and approved the final manuscript.

## References

[B1] Snowden JS Rollinson S Lafon C Harris J Thompson J Richardson AM Jones M Gerhard A Neary D Mann DM Pickering-Brown S Psychosis, C9ORF72 and dementia with Lewy bodies J Neurol Neurosurg Psychiatry 2012 15 10 1031 1032 22832738 10.1136/jnnp-2012-303032

[B2] Rademakers R Neumann M Mackenzie IR Advances in understanding the molecular basis of frontotemporal dementia Nat Rev Neurol 2012 15 8 423 434 22732773 10.1038/nrneurol.2012.117 PMC3629543

[B3] Cooper-Knock J Hewitt C Highley JR Brockington A Milano A Man S Martindale J Hartley J Walsh T Gelsthorpe C Baxter L Forster G Fox M Bury J Mok K McDermott CJ Traynor BJ Kirby J Wharton SB Ince PG Hardy J Shaw PJ Clinico-pathological features in amyotrophic lateral sclerosis with expansions in C9ORF72 Brain 2012 15 Pt 3 751 764 22366792 10.1093/brain/awr365 PMC3286332

[B4] Mioshi E Lillo P Yew B Hsieh S Savage S Hodges JR Kiernan MC Hornberger M Cortical atrophy in ALS is critically associated with neuropsychiatric and cognitive changes Neurology 2013 15 12 1117 1123 23427327 10.1212/WNL.0b013e31828869da

[B5] Lillo P Savage S Mioshi E Kiernan MC Hodges JR Amyotrophic lateral sclerosis and frontotemporal dementia: a behavioural and cognitive continuum Amyotroph Lateral Scler 2012 15 1 102 109 22214356 10.3109/17482968.2011.639376

[B6] Elamin M Bede P Byrne S Jordan N Gallagher L Wynne B O'Brien C Phukan J Lynch C Pender N Hardiman O Cognitive changes predict functional decline in ALS: a population-based longitudinal study Neurology 2013 15 17 1590 1597 23553481 10.1212/WNL.0b013e31828f18ac

[B7] Phukan J Elamin M Bede P Jordan N Gallagher L Byrne S Lynch C Pender N Hardiman O The syndrome of cognitive impairment in amyotrophic lateral sclerosis: a population-based study J Neurol Neurosurg Psychiatry 2012 15 1 102 108 21836033 10.1136/jnnp-2011-300188

[B8] Phukan J Pender NP Hardiman O Cognitive impairment in amyotrophic lateral sclerosis Lancet Neurol 2007 15 11 994 1003 17945153 10.1016/S1474-4422(07)70265-X

[B9] Raaphorst J de Visser M Linssen WH de Haan RJ Schmand B The cognitive profile of amyotrophic lateral sclerosis: a meta-analysis Amyotroph Lateral Scler 2010 15 1–2 27 37 19180349 10.3109/17482960802645008

[B10] Goldstein LH Abrahams S Changes in cognition and behaviour in amyotrophic lateral sclerosis: nature of impairment and implications for assessment Lancet Neurol 2013 15 4 368 380 23518330 10.1016/S1474-4422(13)70026-7

[B11] Abrahams S Leigh PN Goldstein LH Cognitive change in ALS: a prospective study Neurology 2005 15 7 1222 1226 15824350 10.1212/01.WNL.0000156519.41681.27

[B12] Massman PJ Sims J Cooke N Haverkamp LJ Appel V Appel SH Prevalence and correlates of neuropsychological deficits in amyotrophic lateral sclerosis J Neurol Neurosurg Psychiatry 1996 15 5 450 455 8937336 10.1136/jnnp.61.5.450 PMC1074039

[B13] Schreiber H Gaigalat T Wiedemuth-Catrinescu U Graf M Uttner I Muche R Ludolph AC Cognitive function in bulbar- and spinal-onset amyotrophic lateral sclerosis. A longitudinal study in 52 patients J Neurol 2005 15 7 772 781 15742104 10.1007/s00415-005-0739-6

[B14] Abrahams S Leigh PN Harvey A Vythelingum GN Grise D Goldstein LH Verbal fluency and executive dysfunction in amyotrophic lateral sclerosis (ALS) Neuropsychologia 2000 15 6 734 747 10689049 10.1016/s0028-3932(99)00146-3

[B15] Abrahams S Newton J Niven E Foley J Bak TH Screening for cognition and behaviour changes in ALS Amyotroph Lateral Scler Frontotemporal Degener 2014 15 1–2 9 14 23781974 10.3109/21678421.2013.805784

[B16] Abrahams S Executive dysfunction in ALS is not the whole story J Neurol Neurosurg Psychiatry 2013 15 5 474 475 23117493 10.1136/jnnp-2012-303851

[B17] Raaphorst J de Visser M van Tol MJ Linssen WH van der Kooi AJ de Haan RJ van den Berg LH Schmand B Cognitive dysfunction in lower motor neuron disease: executive and memory deficits in progressive muscular atrophy J Neurol Neurosurg Psychiatry 2011 15 2 170 175 20562407 10.1136/jnnp.2009.204446

[B18] Christidi F Zalonis I Smyrnis N Evdokimidis I Selective attention and the three-process memory model for the interpretation of verbal free recall in amyotrophic lateral sclerosis J Int Neuropsychol Soc 2012 15 5 809 818 22676844 10.1017/S1355617712000562

[B19] Kew JJ Goldstein LH Leigh PN Abrahams S Cosgrave N Passingham RE Frackowiak RS Brooks DJ The relationship between abnormalities of cognitive function and cerebral activation in amyotrophic lateral sclerosis. A neuropsychological and positron emission tomography study Brain 1993 15 Pt 6 1399 1423 8293278 10.1093/brain/116.6.1399

[B20] Bak TH Hodges JR Motor neurone disease, dementia and aphasia: coincidence, co-occurrence or continuum? J Neurol 2001 15 4 260 270 11374089 10.1007/s004150170199

[B21] Frank B Haas J Heinze HJ Stark E Munte TF Relation of neuropsychological and magnetic resonance findings in amyotrophic lateral sclerosis: evidence for subgroups Clin Neurol Neurosurg 1997 15 2 79 86 9213049 10.1016/s0303-8467(96)00598-7

[B22] Gallassi R Montagna P Ciardulli C Lorusso S Mussuto V Stracciari A Cognitive impairment in motor neuron disease Acta Neurol Scand 1985 15 6 480 484 4024859 10.1111/j.1600-0404.1985.tb03231.x

[B23] Hanagasi HA Gurvit IH Ermutlu N Kaptanoglu G Karamursel S Idrisoglu HA Emre M Demiralp T Cognitive impairment in amyotrophic lateral sclerosis: evidence from neuropsychological investigation and event-related potentials Brain Res Cogn Brain Res 2002 15 2 234 244 12067696 10.1016/s0926-6410(02)00110-6

[B24] Strong MJ Grace GM Orange JB Leeper HA Menon RS Aere C A prospective study of cognitive impairment in ALS Neurology 1999 15 8 1665 1670 10563610 10.1212/wnl.53.8.1665

[B25] Abrahams S Goldstein LH Al-Chalabi A Pickering A Morris RG Passingham RE Brooks DJ Leigh PN Relation between cognitive dysfunction and pseudobulbar palsy in amyotrophic lateral sclerosis J Neurol Neurosurg Psychiatry 1997 15 5 464 472 9153602 10.1136/jnnp.62.5.464 PMC486852

[B26] Ringholz GM Appel SH Bradshaw M Cooke NA Mosnik DM Schulz PE Prevalence and patterns of cognitive impairment in sporadic ALS Neurology 2005 15 4 586 590 16116120 10.1212/01.wnl.0000172911.39167.b6

[B27] Iwasaki Y Kinoshita M Ikeda K Takamiya K Shiojima T Cognitive impairment in amyotrophic lateral sclerosis and its relation to motor disabilities Acta Neurol Scand 1990 15 2 141 143 2327234 10.1111/j.1600-0404.1990.tb00950.x

[B28] Kato S Hayashi H Yagishita A Involvement of the frontotemporal lobe and limbic system in amyotrophic lateral sclerosis: as assessed by serial computed tomography and magnetic resonance imaging J Neurol Sci 1993 15 1 52 58 8509805 10.1016/0022-510x(93)90089-h

[B29] Mantovan MC Baggio L Dalla Barba G Smith P Pegoraro E Soraru G Bonometto P Angelini C Memory deficits and retrieval processes in ALS Eur J Neurol 2003 15 3 221 227 12752394 10.1046/j.1468-1331.2003.00607.x

[B30] Christidi F Zalonis I Kyriazi S Rentzos M Karavasilis E Wilde EA Evdokimidis I Uncinate fasciculus microstructure and verbal episodic memory in amyotrophic lateral sclerosis: a diffusion tensor imaging and neuropsychological study Brain Imaging Behav 2013 [Epub ahead of print] 10.1007/s11682-013-9271-y 24190400

[B31] Braak H Brettschneider J Ludolph AC Lee VM Trojanowski JQ Del Tredici K Amyotrophic lateral sclerosis–a model of corticofugal axonal spread Nat Rev Neurol 2013 15 12 708 714 24217521 10.1038/nrneurol.2013.221 PMC3943211

[B32] Brettschneider J Del Tredici K Toledo JB Robinson JL Irwin DJ Grossman M Suh E Van Deerlin VM Wood EM Baek Y Kwong L Lee EB Elman L McCluskey L Fang L Feldengut S Ludolph AC Lee VM Braak H Trojanowski JQ Stages of pTDP-43 pathology in amyotrophic lateral sclerosis Ann Neurol 2013 15 1 20 38 23686809 10.1002/ana.23937 PMC3785076

[B33] Bede P Elamin M Byrne S McLaughlin RL Kenna K Vajda A Pender N Bradley DG Hardiman O Basal ganglia involvement in amyotrophic lateral sclerosis Neurology 2013 15 24 2107 2115 24212388 10.1212/01.wnl.0000437313.80913.2c

[B34] Takeda T Uchihara T Arai N Mizutani T Iwata M Progression of hippocampal degeneration in amyotrophic lateral sclerosis with or without memory impairment: distinction from Alzheimer disease Acta Neuropathol 2009 15 1 35 44 19002475 10.1007/s00401-008-0447-2

[B35] Takeda T Uchihara T Mochizuki Y Mizutani T Iwata M Memory deficits in amyotrophic lateral sclerosis patients with dementia and degeneration of the perforant pathway A clinicopathological study J Neurol Sci 2007 15 1–2 225 230 17561122 10.1016/j.jns.2007.05.010

[B36] Mueller SG Schuff N Yaffe K Madison C Miller B Weiner MW Hippocampal atrophy patterns in mild cognitive impairment and Alzheimer's disease Hum Brain Mapp 2010 15 9 1339 1347 20839293 10.1002/hbm.20934 PMC2943433

[B37] Winblad B Palmer K Kivipelto M Jelic V Fratiglioni L Wahlund LO Nordberg A Backman L Albert M Almkvist O Arai H Basun H Blennow K de Leon M DeCarli C Erkinjuntti T Giacobini E Graff C Hardy J Jack C Jorm A Ritchie K van Duijn C Visser P Petersen RC Mild cognitive impairment–beyond controversies, towards a consensus: report of the International Working Group on Mild Cognitive Impairment J Intern Med 2004 15 3 240 246 15324367 10.1111/j.1365-2796.2004.01380.x

[B38] Knopman DS Alzheimer disease biomarkers and insights into mild cognitive impairment Neurology 2013 15 11 978 980 10.1212/WNL.0b013e31828728ac 23390186

[B39] Franko E Joly O Alzheimer's Disease Neuroimaging I Evaluating Alzheimer's disease progression using rate of regional hippocampal atrophy PLoS One 2013 15 8 e71354 23951142 10.1371/journal.pone.0071354 PMC3741167

[B40] Brooks BR Miller RG Swash M Munsat TL World Federation of Neurology Research Group on Motor Neuron D El Escorial revisited: revised criteria for the diagnosis of amyotrophic lateral sclerosis Amyotroph Lateral Scler Other Motor Neuron Disord 2000 15 5 293 299 11464847 10.1080/146608200300079536

[B41] Cedarbaum JM Stambler N Malta E Fuller C Hilt D Thurmond B Nakanishi A The ALSFRS-R: a revised ALS functional rating scale that incorporates assessments of respiratory function. BDNF ALS Study Group (Phase III) J Neurol Sci 1999 15 1–2 13 21 10540002 10.1016/s0022-510x(99)00210-5

[B42] Rascovsky K Hodges JR Knopman D Mendez MF Kramer JH Neuhaus J van Swieten JC Seelaar H Dopper EG Onyike CU Hillis AE Josephs KA Boeve BF Kertesz A Seeley WW Rankin KP Johnson JK Gorno-Tempini ML Rosen H Prioleau-Latham CE Lee A Kipps CM Lillo P Piguet O Rohrer JD Rossor MN Warren JD Fox NC Galasko D Salmon DP Sensitivity of revised diagnostic criteria for the behavioural variant of frontotemporal dementia Brain 2011 15 Pt 9 2456 2477 21810890 10.1093/brain/awr179 PMC3170532

[B43] Aschenbrenner S Lange KW Tucha O RWT: Regensburger Wortflüssigkeits-Test 2000 Göttingen: Hogrefe, Verlag für Psychologie

[B44] Reitan RM Trail Making Test: Manual for administration and scoring 1992 Mesa, Arizona: Reitan Neuropsychology Laboratory

[B45] Härting C Markowitsch H Neufeld H Calabrese P Deisinger K Kessler J WMS-R Wechsler gedächtnistest—revidierte fassung 2000 Bern: Hans Huber

[B46] Helmstaedter C Lendt M Lux S Verbaler Lern-und Merkfähigkeitstest: VLMT; Manual 2001 Göttingen: Beltz-Test

[B47] Osterrieth PA Le test de copie d'une figure complexe Arch Psychol 1944 15 206 356

[B48] Hautzinger M Keller F Kühner C Beck AT Beck Depressions-Inventar: BDI II. Revision 2006 Harcourt Test Services

[B49] Elamin M Phukan J Bede P Jordan N Byrne S Pender N Hardiman O Executive dysfunction is a negative prognostic indicator in patients with ALS without dementia Neurology 2011 15 14 1263 1269 21464431 10.1212/WNL.0b013e318214359f

[B50] Munte TF Troger M Nusser I Wieringa BM Matzke M Johannes S Dengler R Recognition memory deficits in amyotrophic lateral sclerosis assessed with event-related brain potentials Acta Neurol Scand 1998 15 2 110 115 9724008 10.1111/j.1600-0404.1998.tb01728.x

[B51] Simons JS Verfaellie M Galton CJ Miller BL Hodges JR Graham KS Recollection-based memory in frontotemporal dementia: implications for theories of long-term memory Brain 2002 15 Pt 11 2523 2536 12390977 10.1093/brain/awf247

[B52] Rugg MD Yonelinas AP Human recognition memory: a cognitive neuroscience perspective Trends Cogn Sci 2003 15 7 313 319 12860190 10.1016/s1364-6613(03)00131-1

[B53] Yonelinas AP The nature of recollection and familiarity: a review of 30 years of research J Mem Lang 2002 15 3 441 517

[B54] Rugg MD Pearl S Walker P Roberts RC Holdstock JS Word repetition effects on event-related potentials in healthy young and old subjects, and in patients with alzheimer-type dementia Neuropsychologia 1994 15 4 381 398 8047247 10.1016/0028-3932(94)90085-x

[B55] Sarro L Agosta F Canu E Riva N Prelle A Copetti M Riccitelli G Comi G Filippi M Cognitive functions and white matter tract damage in amyotrophic lateral sclerosis: a diffusion tensor tractography study AJNR Am J Neuroradiol 2011 15 10 1866 1872 22016410 10.3174/ajnr.A2658 PMC7966026

[B56] Schuster C Kasper E Dyrba M Machts J Bittner D Kaufmann J Mitchell AJ Benecke R Teipel S Vielhaber S Prudlo J Cortical thinning and its relation to cognition in amyotrophic lateral sclerosis Neurobiol Aging 2014 15 1 240 246 23992619 10.1016/j.neurobiolaging.2013.07.020

[B57] Bede P Bokde A Elamin M Byrne S McLaughlin RL Jordan N Hampel H Gallagher L Lynch C Fagan AJ Pender N Hardiman O Grey matter correlates of clinical variables in amyotrophic lateral sclerosis (ALS): a neuroimaging study of ALS motor phenotype heterogeneity and cortical focality J Neurol Neurosurg Psychiatry 2013 15 7 766 773 23085933 10.1136/jnnp-2012-302674

[B58] Mezzapesa DM Ceccarelli A Dicuonzo F Carella A De Caro MF Lopez M Samarelli V Livrea P Simone IL Whole-brain and regional brain atrophy in amyotrophic lateral sclerosis AJNR Am J Neuroradiol 2007 15 2 255 259 17296989 PMC7977419

